# Structural basis for the inhibition of the SARS-CoV-2 main protease by the anti-HCV drug narlaprevir

**DOI:** 10.1038/s41392-021-00468-9

**Published:** 2021-02-04

**Authors:** Yu Bai, Fei Ye, Yong Feng, Hanyi Liao, Hao Song, Jianxun Qi, George Fu Gao, Wenjie Tan, Lifeng Fu, Yi Shi

**Affiliations:** 1 Savaid Medical School, University of Chinese Academy of Sciences, Beijing, 100049 China; 2 CAS Key Laboratory of Pathogenic Microbiology and Immunology, Institute of Microbiology, Chinese Academy of Sciences, Beijing, 100101 China; 3 NHC Key Laboratory of Biosafety, National Institute for Viral Disease Control & Prevention, Chinese Center for Disease Control and Prevention, China CDC, Beijing, 102206 China; 4 Research Network of Immunity and Health (RNIH), Beijing Institutes of Life Science, Chinese Academy of Sciences, Beijing, 100101 China; 5 Center for Influenza Research and Early Warning (CASCIRE), CAS-TWAS Center of Excellence for Emerging Infectious Disease (CEEID), Chinese Academy of Sciences, Beijing, 100101 China

**Keywords:** Structural biology, Drug screening

**Dear Editor,**

The second wave of the coronavirus disease (COVID-19) pandemic has recently appeared in Europe. Most European countries, such as France, Germany, and Italy, have announced the implementation of a new round of epidemic prevention and control measures. However, no clinical drug or vaccine has been approved for the treatment of COVID-19. The interim results of the solidarity therapy trial coordinated by the World Health Organization (WHO) indicated that remdesivir, hydroxychloroquine, lopinavir/ritonavir, and interferon appear to have little or no effect on the 28-day mortality of hospitalized patients or the hospitalization process of new COVID-19 patients. Therefore, there is an urgent need to develop new drugs against COVID-19.

Many viral protease inhibitors, such as telaprevir, asunaprevir, grazoprevir, simeprevir, and darunavir, have been successfully approved for the treatment of HCV and HIV. For coronavirus, the main protease (M^pro^, 3CL^pro^) and papain-like protease (PL^pro^) are responsible for the digestion of viral polyproteins 1a and 1ab to produce 16 active viral nonstructural proteins. These nonstructural proteins are critical for viral replication and transcription. In particular, M^pro^ cleaves 11 substrate sites of viral polyprotein 1ab and 7 substrate sites of viral polyprotein 1a. Therefore, M^pro^ is recognized as an attractive drug target. The structures of the covalent inhibitors 13b^1^ and N3^2^ when complexed with M^pro^ have been determined at first. Based on the complex structure, structure-based design of the covalent inhibitors 11a and 11b targeting M^pro^ has led to better antiviral activities.^3^ Compared with these preclinical drugs, repurposing approved drugs is a feasible method for emergent treatment of COVID-19 patients. The antineoplastic drug carmofur was screened and it exhibited M^pro^ inhibitory activity. The crystal structure, when complexed with M^pro^, revealed that the carbonyl reactive group of carmofur can covalently bind to catalytic Cys145.^4^ We also found that the anti-HCV drug boceprevir^5^ can effectively inhibit SARS-CoV-2 in Vero cells by targeting M^pro^ with an EC_50_ of 15.57 μM. Further, structural analysis revealed that boceprevir can occupy the substrate-binding pocket of M^pro^ and form a covalent bond with the catalytic Cys145. Narlaprevir is a potent second-generation inhibitor of the HCV NS3 protease based on boceprevir and now is in phase III clinical trials. Unlike boceprevir, narlaprevir is a single isoform and shows an improved pharmacokinetic profile and physicochemical characteristics.

Using an enzyme activity inhibition assay, we found that narlaprevir (Fig. 1a) showed moderate inhibitory activity against SARS-CoV-2 M^pro^, with an IC_50_ value of 16.11 μM (Fig. 1a). To validate the binding of narlaprevir with SARS-CoV-2 M^pro^ and exclude any false-positive results of the enzyme activity inhibition test, we performed isothermal titration calorimetry (ITC) to measure the binding affinity between narlaprevir and SARS-CoV-2 M^pro^. The Kd value of narlaprevir binding with SARS-CoV-2 M^pro^ is 82 μM. In contrast, boceprevir and GC376 have Kd values of 21 μM and 0.46 μM, respectively (Supplementary Fig. S1). These results were consistent with the enzyme activity inhibition assay.

**Fig. 1:**
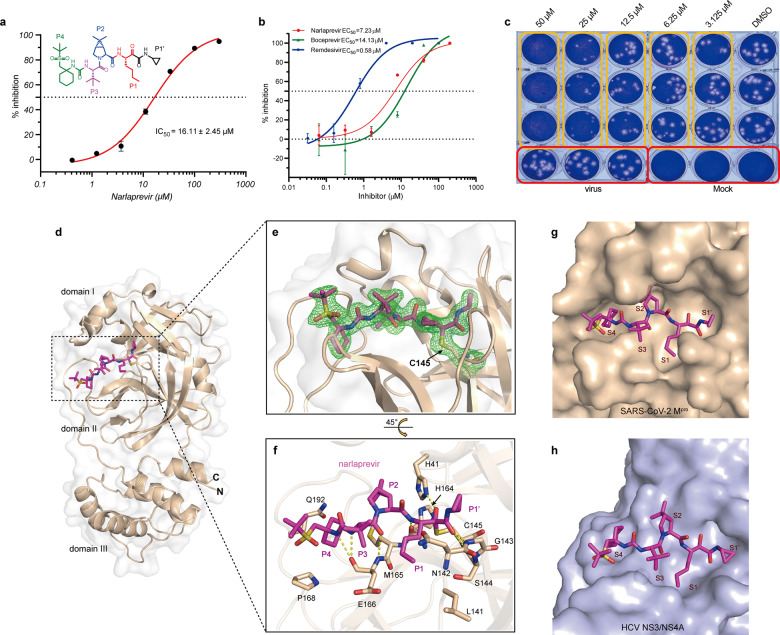
Narlaprevir can inhibit SARS-CoV-2 by targeting M^pro^. **a** Narlaprevir can inhibit the catalytic activity of SARS-CoV-2 M^pro^ in vitro. The cyclopropyl P1’ residue was colored black, the n-butyl P1 residue was colored red, the DMCP P2 residue was colored blue, the tBu P3 residue was colored magenta, the cyclohexyl and appended tBu sulfone P4 residue was colored green in the chemical structure of narlaprevir. **b** The inhibitory effect of narlaprevir on SARS-CoV-2 replication. Remdesivir and boceprevir were used as positive controls. **c** Plaque reduction assay of narlaprevir against SARS-CoV-2. **d** Complex structure of SARS-CoV-2 M^pro^ bound to narlaprevir (magenta). Domains I, II, and III are labeled. **e** Narlaprevir creates a covalent bond with the catalytic residue C145. The 2Fo-Fc electron density map for narlaprevir and the catalytic residue C145 contoured at 1.0 sigma is represented in green. **f** Detailed interactions between narlaprevir and SARS-CoV-2 M^pro^. The amino acids involved are represented as sticks. Hydrogen bond interactions are shown as dashed lines. The P1’-P4 residues were indicated next to the relevant structure. **g** The binding pocket of narlaprevir bound to SARS-CoV-2 M^pro^. M^pro^ is shown in the surface representation. Narlaprevir in the S1’, S1, S2, S3, and S4 positions of the active site of SARS-CoV-2 M^pro^ is labeled. **h** The binding pocket of narlaprevir bound to HCV NS3/4 A serine protease (PDB: 3LON)

Narlaprevir showed an antiviral effect against SARS-CoV-2 with an EC_50_ value of 7.23 μM (Fig. 1b). As a positive control, remdesivir and boceprevir inhibited SARS-CoV-2 replication with EC_50_ values of 0.58 μM and 14.13 μM, respectively. Additionally, narlaprevir exhibited no cytotoxicity in Vero cells at different concentrations up to 200 μM (Supplementary Fig. S2). Treatment with narlaprevir infection demonstrated a dose-dependent inhibitory effect on SARS-CoV-2 plaque formation (Fig. 1c). The plaques were completely inhibited in the presence of 50 μM narlaprevir.

The crystal structure of the M^pro^-narlaprevir complex was determined at 1.78 Å resolution (Supplementary Table S1). The M^pro^ molecule contains three domains and narlaprevir binds to the substrate-binding site located in the cavity between domains I and II of M^pro^ in an extended conformation (Fig. 1d). The unambiguous electron density map shows that narlaprevir binds to the active site of M^pro^ through a C–S covalent bond interaction with catalytic C145 (Fig. 1e and Supplementary Fig. S3). In the M^pro^- narlaprevir complex, residues H41, N142, G143, and H164 form four hydrogen-bonds with the amide backbone of narlaprevir on one side, and residue E166 forms three hydrogen-bonds with narlaprevir on the other side (Fig. 1f). According to the Berger and Schechter nomenclature, narlaprevir can be divided into five moieties, P1–P4 and P1’, as shown in Figs. 1a and 1f. The S1 subsite of M^pro^ was found to be a polarity pocket composed of Phe140, Tyr161, His162, Glu166, and His172. The norleucine moiety at P1 of narlaprevir can fit the S1 pocket shape well (Fig. 1g). The rigid P2 dimethyl-cyclopropyl proline (DMCP) residue lies in the S2 hydrophobic pocket, which is composed of His41, Met49, Met165, Phe181, and Asp187. The hydrophobic P3 tert-butyl (tBu) residue is exposed to solvents in the S3 subsite. The cyclohexyl moiety at P4 is buried deep in the S4 pocket. However, the appended tBu sulfone group is exposed to solvents. In addition, the cyclopropyl moiety at P1’ can also be tolerated by the S1’ pocket due to its small size (Fig. 1g).

Compared with the HCV NS3/4A-narlaprevir complex (Fig. 1h), narlaprevir undergoes a large conformational change to fit the M^pro^ substrate-binding pocket (Fig. 1g). This is similar to boceprevir binding (Supplementary Fig. S4a and S4b). However, narlaprevir has a weaker protease inhibitory activity than boceprevir. The tBu sulfone tail of narlaprevir, which does not appear to favor the S4 pocket of M^pro^, may contribute to the reduction in enzyme potency. In contrast, the tBu sulfone tail and cyclopropyl moiety at P1’ of narlaprevir can increase its biological activity across the cell membrane. This leads to the improved anti-viral activity of narlaprevir over boceprevir against SARS-CoV-2. We also compared the structures of the newly identified compounds complexed with SARS-CoV-2 M^pro^ and found that all target the active site of SARS-CoV-2 M^pro^. These compounds were covalently bound to the catalytic residue Cys145 (Supplementary Fig. S4c–f). Moreover, the Michael acceptor inhibitor N3 is an irreversible covalent inhibitor that shows time-dependent inhibitory activity. In general, the glutamine surrogate ring at the P1 position is essential for the sub-micromolar inhibitory activity in M^pro^ structures complexed with 11a (IC_50_ = 0.053 μM, EC_50_ = 0.53 μM), 13b (IC_50_ = 0.67 μM, EC_50_ = 4-5 μM), N3 (*k*_*3*_/*K*_*i*_ = 11,300 ± 880 M^−1^ s^−1^, EC_50_ = 16.77 μM), and GC376 (IC_50_ = 0.15 μM, EC_50_ = 0.7 μM). The NH residues of the glutamine surrogate ring can form hydrogen-bonds with the carboxyl groups of Glu166 and Phe140. In contrast, the carboxyl group of the surrogate rings can form hydrogen-bonds with His172. Subsites S2, S3, and S4 of SARS-CoV-2 M^pro^ prefer hydrophobic residues. Among them, subsite S2 has a certain flexibility and can accommodate large hydrophobic groups, such as DMCP. The DMCP residue can improve the pharmacokinetic properties and specificity of the entire molecule. Subsite S3 is exposed to solvents. The indole residue of 11a, benzene residue of GC376, isopropyl residue of N3, and tBu residue from both boceprevir and narlaprevir can fit subsite S3. However, none of these groups interacted with the S3 subsite. Whether there are more suitable groups for S3 requires further investigation. For the S4 subsite, the cyclohexyl moiety fits very well and has hydrophobic interactions. This should be considered in future structural optimization. In summary, our work combined with other studies, will provide the structural basis for the optimization and design of more potent drugs to treat SARS-CoV-2 infection.

## Supplementary information

Supplementary Materials

7D1O

7D1O Structure Validation Report

## Data Availability

Coordinates and structural factors have been deposited in the Protein Data Bank under accession code 7D1O.
