# Supplementary material for: Structural basis for the inhibition of the SARS-CoV-2 main protease by the anti-HCV drug narlaprevir
Source: Signal Transduct Target Ther. 2021 Feb 4;6:51. doi: 10.1038/s41392-021-00468-9 (PMC7860160; doi:10.1038/s41392-021-00468-9)
Supplement: Supplementary file 1 — Supplementary Materials [file 41392_2021_468_MOESM1_ESM.docx]

Supplementary Materials for

Structural basis for the inhibition of the SARS-CoV-2 main protease by the anti-HCV drug narlaprevir

Yu Bai, Fei Ye, Yong Feng, Hanyi Liao, Hao Song, Jianxun Qi, George Fu Gao, Wenjie Tan, Lifeng Fu, and Yi Shi

Correspondence to: tanwj@ivdc.chinacdc.cn, fulf@im.ac.cn, shiyi@im.ac.cn

**This PDF file includes:**

Materials and Methods

Figure S1

Figure S2

Figure S3

Figure S4

Table S1

**Materials and Methods**

**Protein expression and purification**

The N-terminus of M^pro^ plays an important role in its enzymatic activity. In particular, only the native N-terminus can keep the optimal activity of the viral protease, because the first Ser residue of one protomer is critical to stabilizing the S1 subsite of its neighbor protomer. So we cloned cDNA sequence encoding residues 3264-3569 of ORF1ab (GenBank: MN908947.3) into the *Nco*I and *Not*I sites of the pET-28b vector (Genscript) with an N-terminal His and SUMO tag. The vector was transformed into *E. coli* strain BL21 (DE3). 0.2 mM isopropyl-β-d-thiogalactopyranoside (IPTG) was added to LB medium at an OD_600_ of 0.6-0.8. The cells were cultured at 16 °C for 18 h. The cells were lysed by sonication and centrifuged for 30 min. The supernatants contained soluble protein were then purified by HisTrap HP 5 ml columns (GE Healthcare). The purified proteins per mg were incubated with 2 μL reconstruction SUMO protease (Beyotime, P2312M) at 30 °C for 2 h after centrifugation and then purified by HisTrap HP 5 ml columns (GE Healthcare) again. The native M^pro^ protein was obtained by Hiload 16/600 Superdex 75 PG column (GE Healthcare) with buffer (10 mM Tris-HCl, 1 mM DTT, 1 mM EDTA, pH 7.5).

**Antiviral compounds**

Narlaprevir (Cat no. HY-10300) and Remdesivir (Cat no. HY-104077) were purchased from MedChemExpress. Bocepreivir (Cat no. T4988) and GC376 (Cat no. T5188) were purchased from TargetMol.

**M^pro^ enzyme activity inhibition test**

30μL 300μM to 0.41 μM narlaprevir in 25 mM Tris buffer (pH=8.0) was mixed with 10μL 100μM FRET substrate firstly. 10μL 50μg/ml M^pro^ was added to the 96 black plate (Greiner, Cat no. 655900) immediately. The relative fluorescence units (RFU) were measured by the SpectraMax Paradigm Muti-Mode Detection Platform (Molecular Devices, USA) with an excitation wavelength of 360 nm and an emission wavelength of 490 nm for 1h. Experiments were performed in triplicate.

**ITC assay**

Measurements were performed on an Affinity ITC LV at 25℃. The protein was dissolved in PBS containing 10% DMSO. The compounds were dissolved into 10 mM with DMSO. The solutions were diluted to 1mM by PBS, and then to 100μM by PBS containing 10% DMSO. An injection series (3.14 μl for the first injection and 10 μl for subsequent injections) was carried out using a 250 μl syringe filled with the test solutions (25 injections at 200 s intervals). Data were analysed using the NanoAnalyze Software v3.11 by using Independent Model.

**Crystallization**

10μL 20mM narlaprevir was mixed with 90μL 8 mg/ml and 12 mg/ml M^pro^ (in 10 mM Tris, 1mM EDTA, 1mM DTT, pH 7.5) respectively. The mixture was incubated at 4°C overnight. 1 μL protein solution was mixed with 1 μL reservoir solution. 100 μL reservoir was added in the middle of a sitting drop vapor diffusion plate. The plate was incubated at 18°C. The M^pro^- Narlaprevir complexes are crystallized in 20% PEG5000, 0.1 M BIS-TRIS (pH 6.5).

**Data collection and structure determination.**

Diffraction data were collected at the Shanghai Synchrotron Radiation Facility (SSRF) BL17U (wavelength, 0.97918 Å). For data collection, the crystals were cryo-protected by briefly soaking in reservoir solution supplemented with 20% (v/v) glycerol before flash-cooling in liquid nitrogen. The dataset was processed with HKL2000 software^26^. The native M^pro^ structure was determined by the molecular replacement method using Phaser^27^ with the previously reported SARS-CoV M^pro^ structure (PDB code, 7C6S), while the complexes were further determined with the solved M^pro^ structure. The atomic models were completed with Coot^28^ and refined with phenix.refine in Phenix^29^, and the stereochemical qualities of the final models were assessed with MolProbity^30^. Data collection, processing, and refinement statistics are summarized in Table S1. All structural figures were generated using PyMOL software (http://www.pymol.org).

**Cells and viruses**

Vero cells were maintained with DMEM culture medium (10% fetal bovine sera, 200 mg/ml streptomycin, and 200 IU/ml penicillin) at 37°C. SARS-CoV-2 virus were provided by China CDC. All the viral experiments were performed in a biosafety level-3 (BLS-3) laboratory.

**Cell viability assay**

100 μL different concentration of narlaprevir solutions were incubated with the overnight cultured Vero cells for 48 h at 37°C with 5% CO_2_. 10 μL CCK8 regent (TargetMol, C0005) was added to the plate directly. After 1h incubation at 37°C, OD_450_ value was measured by a microplate reader (Multiskan FC, Thermo Scientific). Experiments were performed in triplicate.

**In vitro antiviral assays**

0.01 MOI SARS-CoV-2 virus infected overnight cultured Vero cells for 2h. Then the cells were washed with PBS for three times. 100 μL 200μM to 0.064μM narlaprevir, 100μM to 0.032μM remdesivir, and 200μM to 0.064μM boceprevir in DMEM with 1% FBS were incubated with cells for 48 h. The cells were then lysed and the viral RNA was quantified by RT-PCR. Experiments were performed in triplicate.

**Plaque-reduction assays**

100 PFU SARS-CoV-2 viruses in 100 μL infected overnight cultured Vero cells in a 24-well plate for 2 h. After the cells were washed with PBS, 100 μL 200μM to 0.064μM narlaprevir in DMEM containing 2% FBS and 1.2% Avicell were added to the cells. 72h later, the overlay was removed and cells were fixed by 4% polyoxymethylene for 30 min. Finally, the fixed cells were stained with crystal violet working solution.

Supplementary Fig. S1


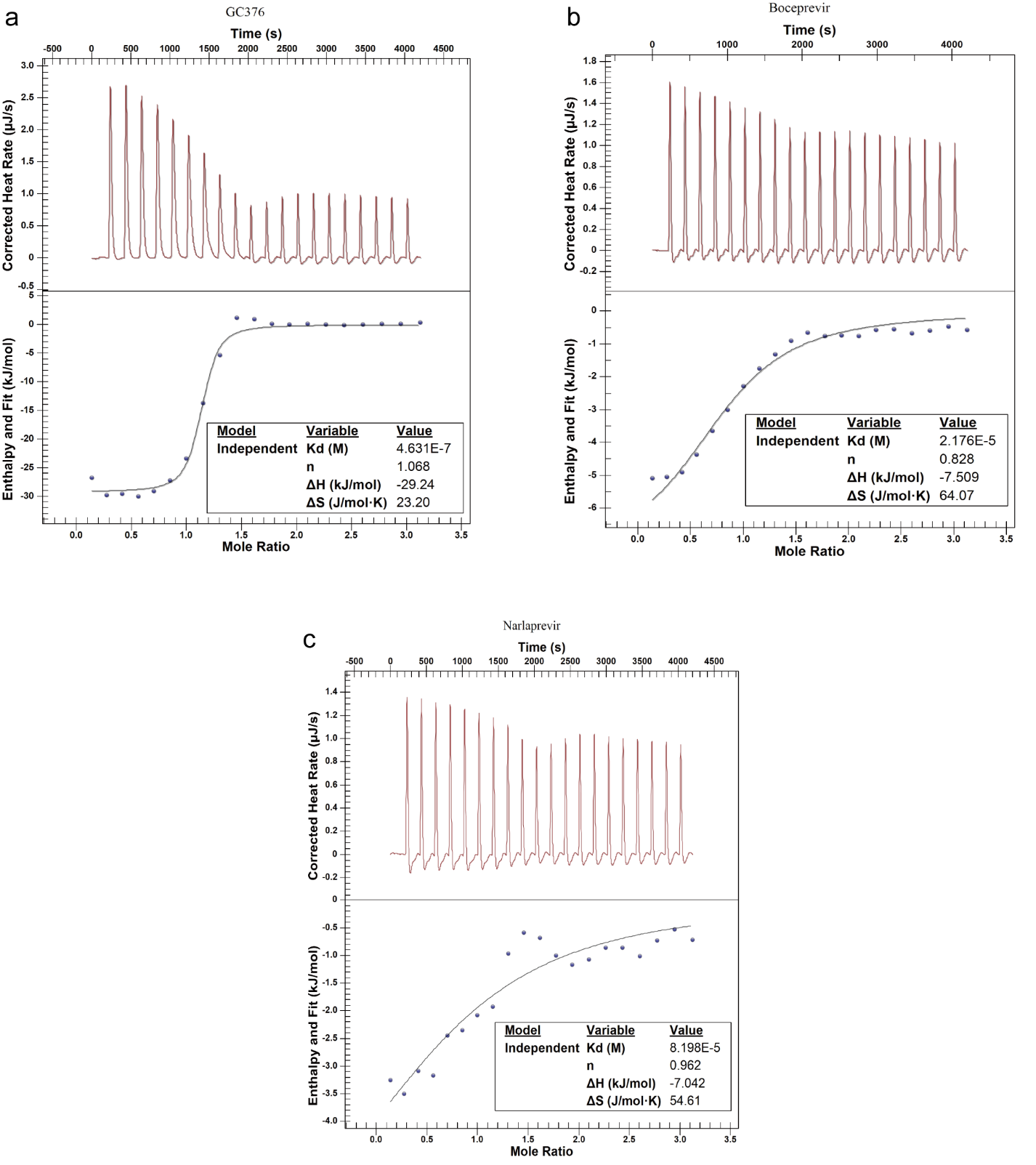


**Figure** **S1** ITC measurements to determine the binding affinity between GC376 (**a**), bocepreivr (**b**), narlaprevir (**c**) and SARS-CoV-2 M^pro^.

Supplementary Fig. S2


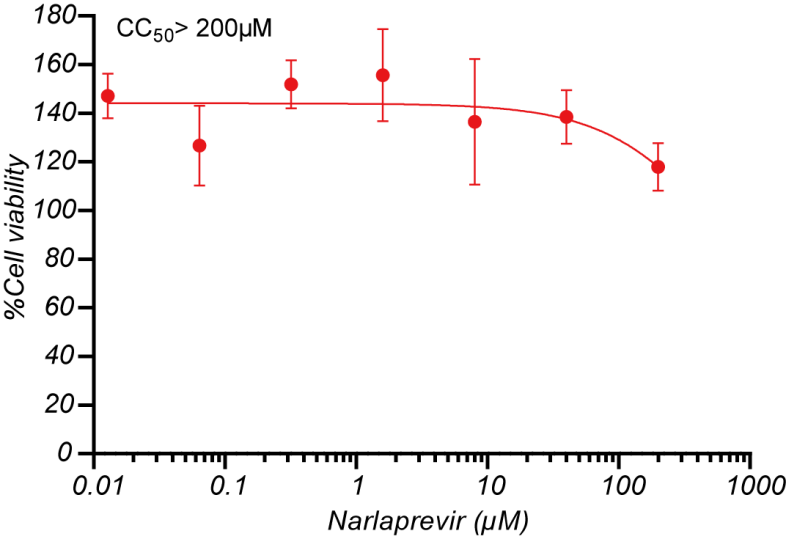


**Figure** **S2** Cytotoxicity assay of narlaprevir on Vero cells was performed by CCK-8 assay. The data are representative of three duplicate wells and the values are expressed in mean ± SD.

Supplementary Fig. S3


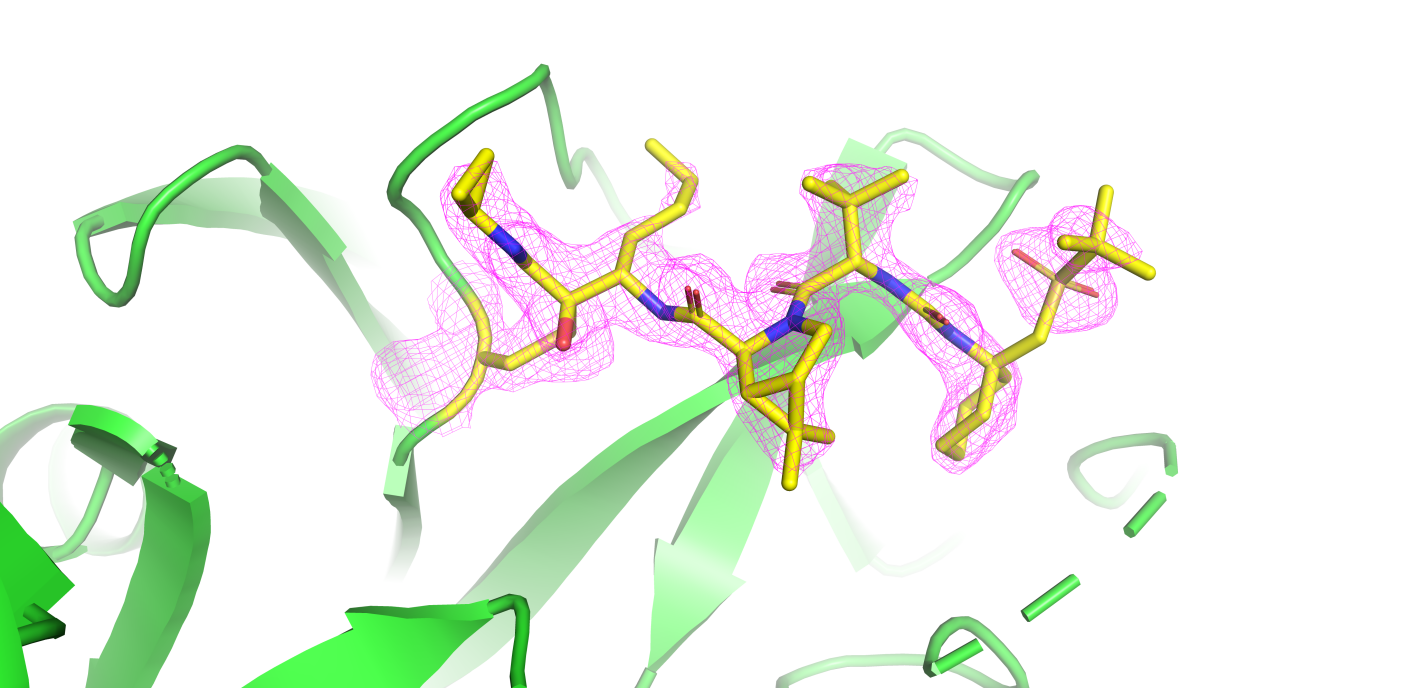


**Figure** **S3** The Fo-Fc electron density map for nalaprevir and the catalytic residue C145 contoured at 1.5 sigma is represented in magenta.

Supplementary Fig. S4


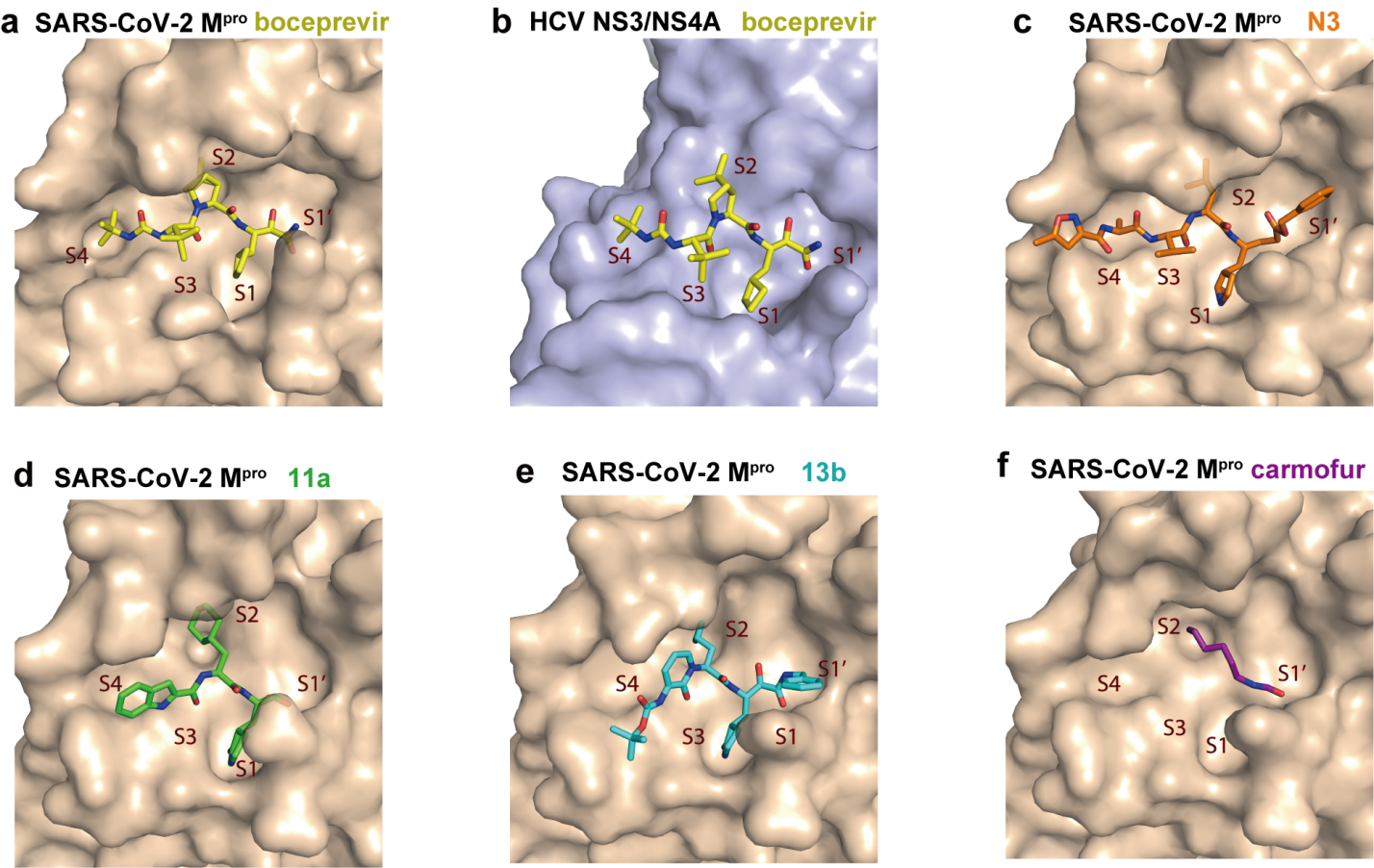


**Figure** **S4** **Comparison of the binding modes of different inhibitors targeting M^pro^ a**, The binding pocket of boceprevir bound to SARS-CoV-2 M^pro^ (PDB: 7C6S). **b**, The binding pocket of boceprevir bound to HCV NS3/4A serine protease (PDB: 2OC8). **c-f**, The binding pockets of 11a (**c**, PDB: 6LZE), 13b (**d**, PDB: 6Y2F), carmofur (**e**, PDB: 7BUY), and N3 (**f**, PDB: 6LU7) bound to SARS-CoV-2 M^pro^ are shown, respectively.

**Supplementary Table S1.**

Diffraction data and refinement statistics

|  | M^pro^-narlaprevir |
| --- | --- |
| **Data collection** |  |
| Space group | C2 |
| Cell dimensions |  |
| *a*, *b*, *c* (Å) | 113.20, 54.05, 45.97 |
| α, β, γ (°)  Wavelength (Å) | 90, 101.61, 90  0.979 |
| Resolution (Å) | 55.44-1.78 (1.78-1.83) |
| *R*_merge_ | 0.048 (0.849) |
| *I* / σ*I* | 16.8 (2.2) |
| CC1/2 | 0.999 (0.741) |
| Completeness (%) | 99.7 (99.8) |
| Redundancy | 6.6 (6.7) |
|  |  |
| **Refinement** |  |
| Resolution (Å) | 55.44-1.78 |
| No. reflections | 25933 |
| *R*_work_ / *R*_free_ | 0.2228/0.2485 |
| No. atoms |  |
| Protein | 2317 |
| Ligand/ion | 49 |
| Water | 172 |
| *B*-factors |  |
| Protein | 40.5 |
| Ligand/ion | 35.5 |
| Water | 48.1 |
| R.m.s. deviations |  |
| Bond lengths (Å) | 0.005 |
| Bond angles (°) | 0.759 |
| Ramachandran plot |  |
| Favored (%) | 96.99 |
| Allowed (%) | 2.68 |
| Outliers (%) | 0.33 |
